# Viruses.STRING: A Virus-Host Protein-Protein Interaction Database

**DOI:** 10.3390/v10100519

**Published:** 2018-09-23

**Authors:** Helen Victoria Cook, Nadezhda Tsankova Doncheva, Damian Szklarczyk, Christian von Mering, Lars Juhl Jensen

**Affiliations:** 1 Novo Nordisk Foundation Center for Protein Research, University of Copenhagen, DK-2200 Copenhagen N, Danmark; helen.cook@gmail.com (H.V.C.); nadezhda.doncheva@cpr.ku.dk (N.T.D.); 2 Center for Non-Coding RNA in Technology and Health, University of Copenhagen, 1870 Frederiksberg C, Danmark; 3 Swiss Institute of Bioinformatics, University of Zurich, 8006 Zurich, Switzerland; damian.szklarczyk@imls.uzh.ch (D.S.); mering@imls.uzh.ch (C.v.M.)

**Keywords:** protein–protein interactions, virus–host interactions, virus bioinformatics, PPI database

## Abstract

As viruses continue to pose risks to global health, having a better understanding of virus–host protein–protein interactions aids in the development of treatments and vaccines. Here, we introduce Viruses.STRING, a protein–protein interaction database specifically catering to virus–virus and virus–host interactions. This database combines evidence from experimental and text-mining channels to provide combined probabilities for interactions between viral and host proteins. The database contains 177,425 interactions between 239 viruses and 319 hosts. The database is publicly available at viruses.string-db.org, and the interaction data can also be accessed through the latest version of the Cytoscape STRING app.

## 1. Introduction

Viruses are well known as global threats to human and animal welfare. Viral diseases such as hepatitis caused by Hepatitis C virus (HCV) and cervical cancer caused by Human papillomavirus (HPV) each cause more than a quarter of a million deaths worldwide each year [1]. Outbreaks also present an economic burden—the 2014 Ebola virus outbreak cost 2.2 billion USD to contain [2], and the annual response to Influenza virus costs five times this amount in medical expenses in the US alone [3]. Climate change and changing land use patterns are causing humans and livestock to be exposed to novel viruses for which there are currently no vaccines or antiviral drugs [4]. This trend will continue as the habitats of vectors that carry arboviruses expand [5], and as humans continue to come into contact with wildlife, creating opportunities for zoonosis [6].

As obligate intracellular parasites, viruses act as metabolic engineers of the cells they infect as they commandeer the cell’s protein synthesis mechanisms to replicate [7]. Thus, it is important to study their interactions with host cells in order to understand their biology, especially how their disruption of the host protein–protein interaction (PPI) network causes disease [8]. Antiviral drugs have been highly effective at preventing the progression of HIV infection to AIDS [9]; however, the effectiveness of antiviral drugs can decrease over time due to the development of drug resistant viral strains [10,11,12,13]. A more complete understanding of the host–virus protein–protein interaction network provides more potential viral drug targets, and also enables alternative strategies such as targeting host proteins to attenuate viral infection [14]. When available, vaccines are very effective at preventing diseases caused by viruses [15]; however, vaccines are not available for all viruses, including HIV-1 and HCV, and a universal Influenza vaccine is still elusive [16]. The development of modern vaccines such as subunit vaccines, which can be administered to immunocompromized patients, and which eliminate the chance that the vaccine could revert to an infectious virus [17], also hinges on understanding the protein–protein interactions between viruses and their hosts.

Novel protein–protein interaction information is disseminated primarily in the scientific literature, but it is not always organized in ways that make it easy to find, access, or extract. Databases such as VirusMentha [18] and HPIDB [19] make strong efforts to organize virus–virus and virus–host PPIs into databases, where this information is available in an easily parsable format. However, with the volume of the biomedical literature growing exponentially at 4% per year [20], it is not feasible for human curators to thoroughly review all new publications to add any new evidence to curated databases [21]. Automated text-mining methods are thus required to get a comprehensive picture of what is already known about the viruses we study.

We have expanded the popular database STRING [22] to include intra-virus and virus–host PPIs. The STRING database has been in constant development for 15 years, and the current version includes protein interaction data for over 2000 species; however, all of the interactions are exclusively intra-species. In this work, for the first time, we include cross species interactions into the STRING database. The PPIs reported by STRING represent functional associations between proteins. These interactions are not limited to physical interactions, and may also include interactions such as transcription factor binding, or the interaction may represent the fact that the associated proteins appear in the same biological pathway. In this paper, the terms “interaction” and “PPI” are used to refer to functional associations. STRING combines many different sources (channels) of information to give a confidence score that measures the probability that the interaction is true. In a similar fashion, we provide virus-related probabilistic interaction networks derived from text mining and experiment channels.

## 2. Materials and Methods

### 2.1. Text Mining Evidence

Text mining for virus species and proteins was conducted using the dictionary-based software described in [23], the same tool that is used for the STRING text mining pipeline. The dictionary for virus species was constructed from NCBI Taxonomy [24], with additional synonyms taken from Disease Ontology [25] and the ninth ICTV report on virus taxonomy [26] to give 173,767 names for 150,885 virus taxa. The virus protein dictionary was constructed from the 397 reference proteomes that were present in UniProt [27] on 31 August 2015. All virus protein names and aliases were expanded following a set of rules to generate variants. This gave 16,580 proteins with 112,013 names. This dictionary was evaluated against a benchmark corpus of 300 abstracts that were annotated by domain experts [28]. The host species and protein dictionaries were identical to those used during the text mining for STRING 10.5 [22]. The text mining was conducted over a corpus that contained the more than 26 million abstracts in PubMed [20], and more than 2.2 million full text articles. The text mining software tags locations at which a protein has been referred to by any of the names that represent it, and then it calculates co-occurrence scores for pairs of proteins. The co-occurrence scores are normalized to account for the fact that some proteins are mentioned very often [29]. The interactions found by this method represent functional associations between the identified proteins.

### 2.2. Experimental Evidence

Experimental data for virus–virus and virus–host PPIs was imported from BioGrid [30], MintAct [31], DIP [32], HPIDB [19] and VirusMentha [18]. These virus–host interactions were scored and then benchmarked against a gold standard set derived from the Kyoto Encyclopedia of Genes and Genomes, (KEGG). This creates a mapping between the number of interacitons mentioned in a study and the probability that they are true interactions according to the benchmark set [33]. The interactions found by this method represent physical interactions.

### 2.3. Transfer Evidence

**Figure 1:**
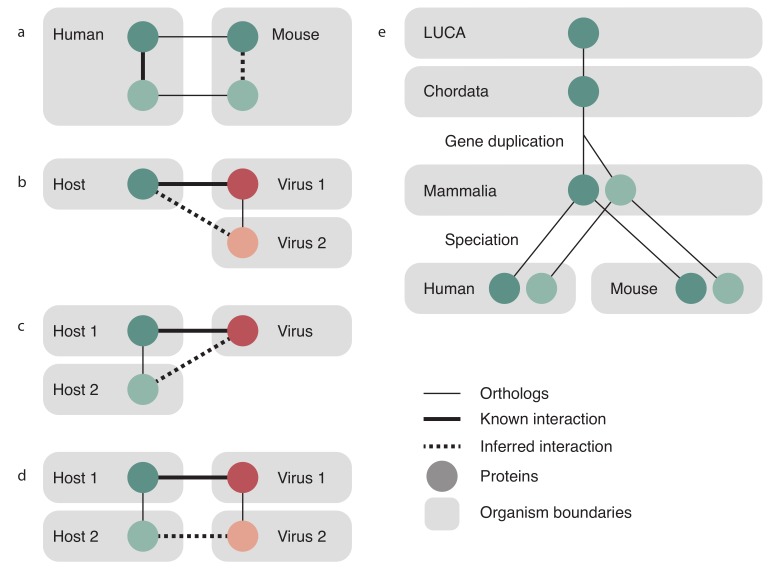
Orthologous relationship transfer in Viruses.STRING. STRING intra-species interactions are transferred between organisms as shown in (**a**): an interaction between two proteins in species 1 (solid thick line) is transferred to two orthologous proteins in species 2 (dashed line). Orthologous relationships are indicated by solid thin lines. This relationship is identical to transferring an interaction between two virus proteins of one virus species to two orthologous proteins in another virus species. Cross species interactions are handled as one of three cases—same host to closely related virus (**b**), the same virus to closely related host (**c**), or both a new host and new virus at the same time (**d**). Note that we transfer only to already known virus–host pairs and that we are not predicting new virus–host relationships via orthology transfer; (**e**) shows the evolutionary history of a gene that underwent a gene duplication event after the last common ancestor of Chordata, but prior to the last common ancestor of mammals. There was subsequently a speciation event that resulted in the duplicated gene being present in both human and mouse. Orthology groups can be read by following the lines up the tree—at the level of Mammalia, the light and dark genes are in separate orthology groups, but at higher levels, they are in the same orthology group.

Orthologous relationships were used to transfer interactions following the same protocol that STRING uses, which is briefly described here and fully described in [29]. Both virus and host orthology relations were taken from EggNOG 4.5 [34]. STRING transfers an interaction between two proteins of the same species to two orthologous proteins in another species as is shown in Figure 1a, and exactly the same was done to also transfer virus–virus PPIs. For transfer of a host–virus PPI, three cases are possible and are illustrated in Figure 1b–d. The known interaction between a virus protein and host protein could be transferred to an orthologous virus protein in a different virus species (panel b), to an orthologous host protein in a different host (panel c), or both cases simultaneously, to both a new virus and a new host (panel d). Transfer is made only between viruses and the hosts they are known to infect; we do not predict new host–virus pairs based on orthology.

The score assigned to the transfer of evidence is a scaled fraction of the score for the original interaction, proportional to how distant the recipient species is. Distance here is calculated as a self normalized bit score, i.e., the bit score of the alignment of the pair of proteins divided by the bit score of the self-alignment of the shorter of the two proteins. Paralogs are considered to be orthologs for the purposes of calculating the score at levels lower than the gene duplication, but at levels higher than the gene duplication, the score is discounted. For virus–virus transfer, the paralog discount is calculated as the reciprocal of the product of the number of proteins of that species in the donor and target orthology groups. For the case of transferring a virus–host PPI, the paralog discount is calculated as the reciprocal of the product of the number of host proteins in the host orthology group and the number of viral proteins in the viral orthology group.

Figure 1e shows three orthology levels (Last universal common ancestor (LUCA), Chordata, Mammalia) and illustrates a gene that has duplicated after Chordata but prior to the last common ancestor of all mammals. Furthermore, there has been a speciation event after Mammalia, separating humans and mice into separate species. At the level of Mammalia, these two proteins are placed in different orthology groups, so any interactions that occur with the darker protein will not be transferred to interaction evidence for the lighter protein. However, at the level of Chordata, the light and dark proteins are in the same orthology group and so will both contribute their confidence to the resulting interaction. The contribution of these two proteins will be penalized since they are paralogs at a lower level. Although it is illustrated here for cellular organisms, this process is also applied to transfers involving viral orthology groups. The final transfer scores are then benchmarked the same way as the scores for the other channels.

## 3. Results

We were able to identify 177,425 protein–protein interactions for 239 viruses. Seventy-seven of these are human viruses, and the remainder infect a total of 318 other hosts. These interactions include more hosts than viruses since many viruses, such as arboviruses, infect multiple hosts. The median number of proteins coded for by these viruses is nine, with 24 viruses coding for more than 100 proteins, and 74 viruses coding for less than nine proteins. The majority of all types of interactions are between viruses and their hosts (as opposed to being intra-virus interactions), due to viral genomes encoding many fewer proteins than their host genomes and thus having fewer potential interactions. In this and the subsequent analysis, interactions are counted per channel, disregarding their scores. Excluding orthology transfer, 89% of the interactions are derived from text mining evidence, and the remaining untransferred evidence comes from curated experimental databases. For 154 viruses, representing 19.8% of all evidence in the database, only text mining evidence is present. This means that, although the interaction has not been curated into databases, we have found a protein from this virus mentioned together in the literature with either other proteins from this virus, or a protein from a host that this virus infects. For 77 viruses, representing 77.4% of all evidence, all of the experimental evidence is also supported by text mining evidence. The remaining eight viruses, representing 2.8% of evidence, have more experimental evidence than text mining, and likely represent opportunities to improve the text mining dictionaries. Despite the large efforts of database curators, the vast wealth of information on PPIs is accessible only in the literature. Furthermore, in addition to physical interactions, text mining will also uncover functional associations such as genetic interactions. As such, text mining provides a very important contribution to this database.

The top GO terms that are enriched in the set of 1835 human proteins that interact with any virus protein with a confidence of 0.5 or greater are shown in Table 1. The fact that this list includes terms such as viral process, protein binding and cell surface receptor signaling pathways provides a sanity check that the human protein partners in the found interactions are valid.

Orthology transfer gives a 2.7 times increase in the number of interactions with text mining results being more readily transferred than experimental results. A handful of well studied viruses (EBV, HIV-1, Influenza A) are the subjects of high-throughput studies that make up the bulk of the interactions in curated experimental databases. These viruses happen to have few close relatives (e.g., HIV, Influenza A), and infect a limited number of hosts (EBV, HIV), which is why their PPIs are not as readily transferred via orthology as interactions found by text mining for other virus proteins. Viruses with many close relatives will have more populated orthology groups, and thus more candidates to transfer evidence from and to. At the level of ssRNA viruses, the maximum size of any orthology group containing an Influenza A protein is 5, and at the level of retrotranscribing viruses the same for any HIV protein is 9. This is in contrast to the viruses that receive the most experimental transfer data, which have 193 (Swinepox virus), 16 (Canine oral papillomavirus) and 30 (Murine cytomegalovirus) proteins in their most populated orthology group. The viruses that receive the most text mining transfer data are Gallid herpesvirus, Murine cytomegalovirus and Equine herpesvirus 2, which at the level of dsDNA have maximum orthology group sizes of 62, 30 and 62 proteins, respectively.

More than half (55%) of pre-transfer evidence relates to human viruses. However, evidence transferred to humans comprises only 26% of all transferred experimental evidence and 18% of all transferred text mining evidence, which implies that the majority of transferred evidence is to a new host (panel c or d in Figure 1). This is due to the fact that gene duplication events occur less frequently in viruses compared to their host organisms [34], and additionally also because the viral taxonomic tree has overall been sequenced much less densely than the tree for cellular organisms, even though the viral tree has been extensively sequenced around specific human viruses [35]. In all, this makes potential transfer partners rarer for transfer between viruses than between hosts.

**Figure 2:**
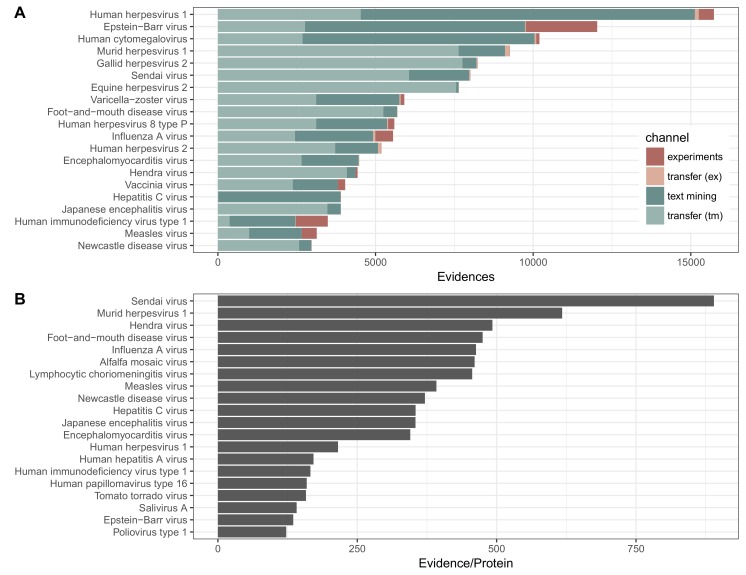
Intra- and intervirus interactions in viruses.STRING by species. (**A**) distribution of experimental (red) and text mining (green) interactions present in the database, further divided into direct (dark red and dark green) and transferred (light green and pink) evidence. Data is shown for the 20 viruses with the most evidence. Evidence may be between two proteins of one virus species, or between a virus protein and a host protein. Evidence is counted as interaction pairs per channel, such that an interaction that is supported by three channels will be counted as 3 evidences. The sources of evidence that is transferred may originate from experimental (abbreviated ex in the figure) or from text mining (tm) data; (**B**) amount of evidence normalized by the number of proteins coded for by that virus.

The distribution of interactions for the 20 viruses with the most interactions is shown in Figure 2. The viruses with the largest number of intra-virus interactions include the relatively large double-stranded DNA Herpesviruses and well studied RNA viruses including Influenza and HIV. The same viruses also show the highest proportion of interactions from the experimental channel. An example of two viruses that share interactions based on orthology transfer are human and murine cytomegalovirus (HCMV and MCMV, respectively). The majority of the evidence for HCMV is direct evidence, and conversely, the majority of evidence for MCMV is evidence from transfer, which has come from interactions with HCMV.

The virus–virus and virus–host PPI networks are made publicly accessible as a resource which is available at viruses.string-db.org. The data can be browsed online, downloaded from the website, or accessed through the REST API. Furthermore, the data can also be imported into Cytoscape directly [36] using the STRING Cytoscape app [22].

### 3.1. Utility and Examples

#### 3.1.1. Web Interface

The Viruses.STRING website enables three variants of protein search: for the complete set of proteins in a virus, for a single protein in a virus, or for multiple proteins in a virus. Since most viral genomes encode only a small number of proteins (the viruses included in the database have a median of nine proteins), they can easily be displayed in a network together with the most strongly interacting host proteins.

The network interface has a similar appearance to STRING, but the visual styling has been modified to be more flat. The nodes in the network are coloured only based on their origin, either as viral proteins (brick red) or as host proteins (blue-green slate).

As is possible on the main STRING site, the viruses.STRING web interface provides more information about each protein, which is accessed by clicking on the node. Similarly, clicking on any edge displays a summary of the information that contributes to that interaction, and provides links to further inspect the evidence from each channel. Text mining evidence shows highlighted phrases from relevant publications, whereas experiments’ evidence shows the specific database and publication from which it was obtained.

#### 3.1.2. Example: HIV-1

**Figure 3:**
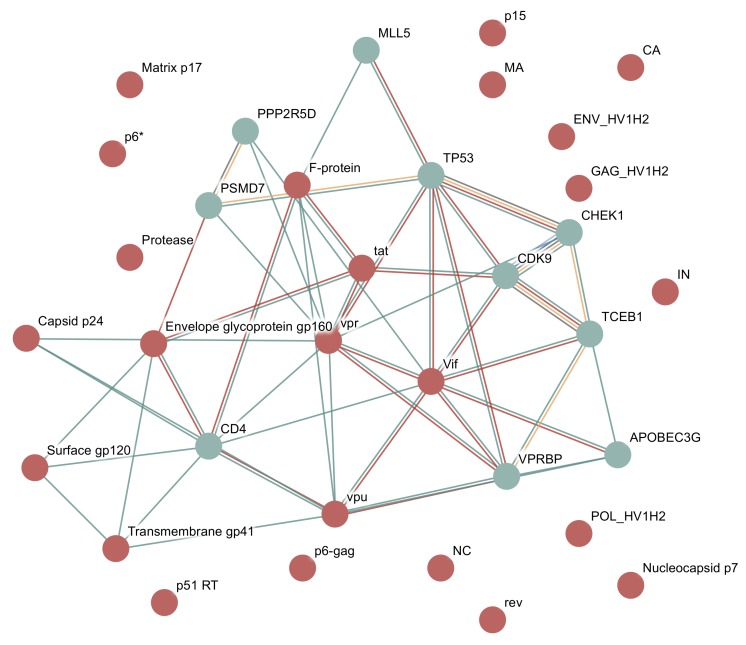
HIV-1 and *Homo sapiens* interaction network in viruses.STRING. HIV-1 and *Homo sapiens* interaction network downloaded as a vector image from viruses.STRING. The type of interaction is indicated by edge colour—green: text mining, red: experiments. Additional edge types can be found between host proteins, and come from the STRING database—yellow orange: pathway databases, light blue: protein homology, dark blue: gene co-occurrence, black: co-expression, light green: gene neighbourhoods, pink: gene fusions (last two not present in figure).

In this example, we will query for all proteins present in Human Immunodeficiency virus type 1. If the host field on the search page is left empty, the server will auto detect the host species with the most interactions with the specified virus, in this case, humans. An interaction network will then be shown for the virus proteins and for the 10 human proteins that have the highest interaction scores with these virus proteins, as shown in Figure 3. By default, only interaction scores with a confidence score greater than 0.4 will be shown, the same as the main STRING site. This confidence can be changed using the interface.

HIV-1 consists of 19 proteins, 10 of which are cleaved from three polyproteins. The polyproteins are translated as a single long protein, and then the long polyprotein is cleaved by the viral protease into functional protein units. The database includes 24 proteins as it includes some partial cleavage products, such as both gp160 and gp120, which is cleaved from gp160.

#### 3.1.3. Cytoscape STRING App

The Viruses.STRING interaction data can also be queried from the Cytoscape STRING app. This requires version 3.6 of Cytoscape or greater and version 1.4 of the STRING app or greater, which is available for free in the Cytoscape app store (http://apps.cytoscape.org/apps/stringapp).

The STRING app allows for more flexible queries than the Viruses.STRING website, such as choosing specific additional host proteins to be included in the network, and displaying multiple hosts and multiple viruses in the same network. In addition to the Viruses.STRING interaction data, the app automatically fetches node and edge information, which can be used for further analysis. The former includes the protein sequence for host and virus nodes, subcellular localization data from the COMPARTMENTS database for human proteins, and tissue expression data taken from the TISSUES database for human, mouse, rat and pig proteins [37]. Edge information includes the combined confidence score from all channels as a probability that the interaction is true.

**Figure 4:**
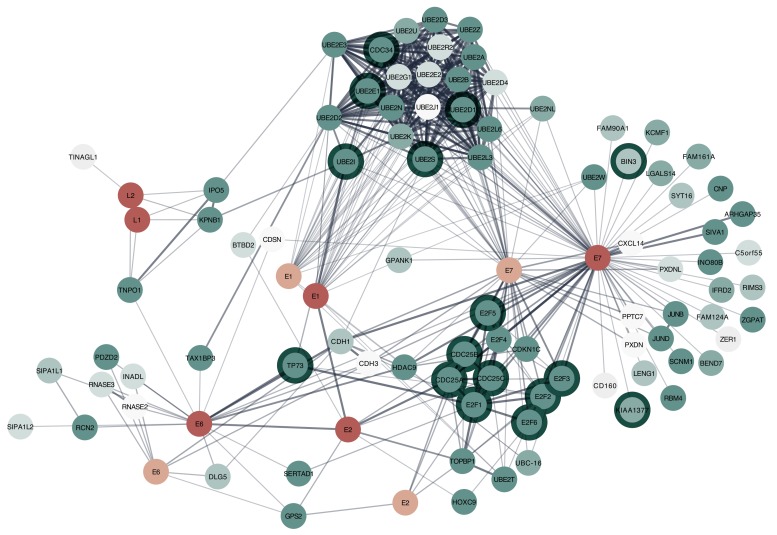
HPV and *Homo sapiens* interaction network in Cytoscape. Proteins from Human Papillomavirus type 16, and HPV type 1a with their human protein interaction partners. Virus proteins are coloured according to their species (dark red: HPV 16, light red: HPV 1a). The human proteins are coloured in shades of green with darker colours showing a stronger association with the nucleus. The dark halos around human proteins are those that are associated with the GO term for cell cycle. The HPV E6 and E7 proteins are known to interfere with the cell cycle. This analysis shows some of the data exploration and visualization flexibility that is easily possible within Cytoscape.

Figure 4 illustrates the combined interaction network for HPV 16 and HPV 1a proteins with the top 50 human proteins they interact with ranked by combined interaction score. Since HPV is known to disrupt the cell cycle [38], many of the proteins that interact with E6 and E7 are associated with the nucleus and the GO term for cell cycle. A tutorial to reproduce this network in Cytoscape is available at http://jensenlab.org/training/stringapp/.

## 4. Discussion

The Viruses.STRING database provides a single unified interface to virus–virus and host–virus PPIs from text mining and many experimental sources. With a simple web interface, the database can easily be queried to immediately retrieve the interaction partners for a protein of interest, and the corresponding evidence can be inspected. The Cytoscape STRINGapp, although it requires software to be installed, provides more versatility than the website, and can handle much larger networks—up to at least as large as the human interaction network. This provides the researcher with more opportunities to answer interesting biological questions about viruses and their hosts. For example, the virus–host network could potentially be used to select candidate host proteins as drug targets to inhibit viral infection, possibly by repurposing existing drugs. This approach would likely generate less viral resistance to the drug since the host protein is being targeted, instead of a viral protein that can mutate easily [14,39].

As this is the first iteration of Viruses.STRING, there are currently some limitations to the data. The virus data is provided only at the species level, with the exception of Dengue types 1–4, even though there is some evidence that different influenza strains show differential protein interactions [40]. This fine grained resolution will be added in a future version for those viruses where sufficient data is available, such as Influenza A.

Text mining reveals many more virus–host PPIs in the literature than have been collected into databases. The text mining gives good precision and recall for virus species, and good precision for virus proteins [28]. However, the method performs less well for virus proteins in terms of recall, meaning that many interactions may still be missed by this approach [28].

Both text mining and experimental evidence is transferred via orthology using the system designed for STRING for cellular organisms. Since viruses have a much higher mutation rate than cellular organisms, this transfer method may over-discount contributions from viruses that appear to be distant relatives but that have diverged relatively recently. This will result in lower transfer scores for viruses than we would see between cellular organisms that have diverged for the same amount of time.

Just as a having a broader view of intra-species PPIs has provided a deeper understanding of cellular function [41], having a similar understanding between pathogens and their hosts will provide new information to combat clinically and economically relevant viral infections and diseases.

## Figures and Tables

**Table 1:** Top gene ontology (GO) terms by false discovery rate (FDR) adjusted *p*-value that are enriched for the human proteins that interact with any virus protein.

Number of Genes	FDR *p*-Value	GO Term
549	2.28E-104	positive regulation of macromolecule metabolic process
718	5.96E-93	positive regulation of cellular process
452	3.02E-91	cell surface receptor signaling pathway
758	7.87E-91	protein binding
779	1.23E-90	positive regulation of biological process
539	1.61E-87	positive regulation of cellular metabolic process
459	3.86E-84	multi-organism process
277	4.56E-82	innate immune response
468	5.27E-82	carbohydrate derivative binding
595	1.99E-81	response to stress
240	2.07E-81	multi-organism cellular process
254	2.4E-81	regulation of immune response
239	2.55E-81	viral process
583	4.17E-81	regulation of response to stimulus

## Abbreviations

The following abbreviations are used in this manuscript:

HCMV	Human cytomegalovirus
HIV	Human immunodeficiency virus
MCMV	Murine cytomegalovirus
PPI	Protein–protein interaction
